# Quantitative Proteomic Analysis Reveals the Key Molecular Events Driving *Phaeocystis globosa* Bloom and Dissipation

**DOI:** 10.3390/ijms232012668

**Published:** 2022-10-21

**Authors:** Shu-Fei Zhang, Bei-Bei Han, Rong-Jun Shi, Feng-Xia Wu, Yi-Yong Rao, Ming Dai, Hong-Hui Huang

**Affiliations:** 1 Guangdong Provincial Key Laboratory of Fishery Ecology and Environment, South China Sea Fisheries Research Institute, Chinese Academy of Fishery Sciences, Guangzhou 510300, China; 2 Southern Marine Science and Engineering Guangdong Laboratory (Guangzhou), Guangzhou 511485, China

**Keywords:** *Phaeocystis globosa*, colony, harmful algal bloom, quantitative proteomics, TMT

## Abstract

*Phaeocystis globosa* is a marine-bloom-forming haptophyte with a polymorphic life cycle alternating between free-living cells and a colonial morphotype, that produces high biomass and impacts ecological structure and function. The mechanisms of *P. globosa* bloom formation have been extensively studied, and various environmental factors are believed to trigger these events. However, little is known about the intrinsic biological processes that drive the bloom process, and the mechanisms underlying *P. globosa* bloom formation remain enigmatic. Here, we investigated a *P. globosa* bloom occurring along the Chinese coast and compared the proteomes of in situ *P. globosa* colonies from bloom and dissipation phases using a tandem mass tag (TMT)-based quantitative proteomic approach. Among the 5540 proteins identified, 191 and 109 proteins displayed higher abundances in the bloom and dissipation phases, respectively. The levels of proteins involved in photosynthesis, pigment metabolism, nitrogen metabolism, and matrix substrate biosynthesis were distinctly different between these two phases. Ambient nitrate is a key trigger of *P. globosa* bloom formation, while the enhanced light harvest and multiple inorganic carbon-concentrating mechanisms support the prosperousness of colonies in the bloom phase. Additionally, colonies in the bloom phase have greater carbon fixation potential, with more carbon and energy being fixed and flowing toward the colonial matrix biosynthesis. Our study revealed the key biological processes underlying *P. globosa* blooms and provides new insights into the mechanisms behind bloom formation.

## 1. Introduction

*Phaeocystis globosa* is a marine phytoplankton that causes blooms in coastal seas of temperate and tropical regions [1]. It has a unique polymorphic life cycle consisting of free-living cells that are generally 3–10 μm in diameter and a colonial morphotype that can reach up to 3 cm in diameter [2,3]. When *P. globosa* blooms, the colonies produce massive amounts of biomass and dominate the phytoplankton communities, thus impacting food web structures and aquaculture, threatening the safety of coastal nuclear power plants, and influencing global biogeochemical cycles and even climate regulation [4,5,6]. In recent years, *P. globosa* has caused harmful algal blooms (HABs) almost every year and has become the primary problematic species in Chinese coastal waters [1,7].

Previous studies have demonstrated that *Phaeocystis* species differ from other marine phytoplankton in their ability to form large gelatinous colonies with a unique colony matrix, which plays a significant role in their success and uniqueness in the environment [8]. This colonial morphotype protects colonial cells from zooplankton grazing, pathogen infection and bacterial colonization [9,10,11]. Moreover, the colony matrix can act as an energy and nutrient reservoir and gives *Phaeocystis* a competitive advantage against other species when resources are scarce and variable [12,13]. Therefore, assessing the triggering factors of colony formation and disruption is of prime importance in understanding the mechanisms behind *Phaeocystis* blooms.

Temperature is one of the most important environmental factors that triggers outbreaks and shapes the field distributions of marine phytoplankton. *P. globosa* grows on a temperature spectrum spanning from 2 to 30 °C, and optimally at 15 to 20 °C [14,15]. Molecular analysis has revealed that a higher temperature decreases synthesis and increases degradation of glycosaminoglycan (GAG), which comprises the *Phaeocystis* colony matrix, thus inhibiting GAG accumulation and colony formation [16]. Irradiance is another determinant of colony formation, since the newly synthesized carbon inside the mucilaginous matrix accounts for more than half of the total photoproducts [15]. Moreover, irradiance controls the precipitation and accumulation of trace metals, such as Mn and Fe, in colonies via a photosynthesis-regulated process of pH and oxygen concentration [13]. Interestingly, pH and oxygen fluctuations have been further suggested to control colony buoyancy, which confers an advantage when accessing nutrients [17,18]. Regarding nutrients, previous studies confirmed that *P. globosa* competes well for nitrogen but poorly for inorganic phosphate in light-saturated conditions [19]. Nitrogen, especially nitrate, is an important trigger and regulator of *P. globosa* blooms, according to both field investigations and mesocosm experiments [20,21,22]. Overall, *P. globosa* bloom outbreaks are a combined result of the environmental conditions that induce colony formation and the strong competitiveness derived from the colony matrix structure. However, little is known about the intracellular biological processes that drive the bloom process, and the mechanisms behind *P. globosa* blooms remain unclear.

Previously, a *P. globosa* bloom occurred and lasted for approximately half a month in Dapeng Bay off the southern coast of China. Field investigation showed that the morphology and distribution of *P. globosa* colonies were obviously different as the bloom developed. These variations are likely associated with some intrinsic biological processes, and the proteins involved in these processes should contain crucial information related to bloom formation, since proteins are the actual executants of life functions. Therefore, in this study, we selected in situ *P. globosa* colonies from the bloom and dissipation phases, and compared their proteomes using a tandem mass tag (TMT)-based quantitative proteomic approach. Based on these findings, the functions of differentially abundant proteins between these two phases were characterized, and key biological processes that might be associated with bloom development were analyzed. The purpose of this study was to identify the key biological processes driving *P. globosa* blooms and to reveal the molecular mechanisms underlying bloom formation.

## 2. Results

### 2.1. Colony Density and Vertical Distribution

**Figure 1:**
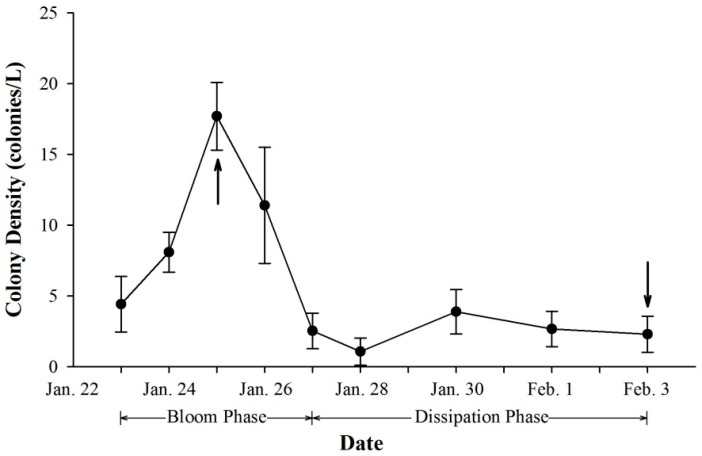
Dynamics of colony density during the *Phaeocystis globosa* bloom period. Values are reported as the means of triplicates with standard deviations. Arrows represent the sampling of proteomic study.

According to the field investigation, colony density increased sharply in the early blooming stage and reached the highest level of approximately 18 colonies/L on the third day (Figure 1). In this period, the colony morphology was spherical and intact, with varying sizes of colonies distributed throughout the whole water column (Figure S1A,B). The colony density then decreased in the following two days and remained at a relatively low level until the end of the investigation (Figure 1). Most colonies settled on the sea bottom, with only a few colonies scattered in surface water (Figure S1C–F). As the bloom continued, the senescent colonies lost the spherical morphotype and became more fragile. On the last day of investigation, most colonies ruptured into fragments with massive debris floating at the bottom (Figure S1E,F).

### 2.2. Dynamics of Physical and Chemical Parameters

**Figure 2:**
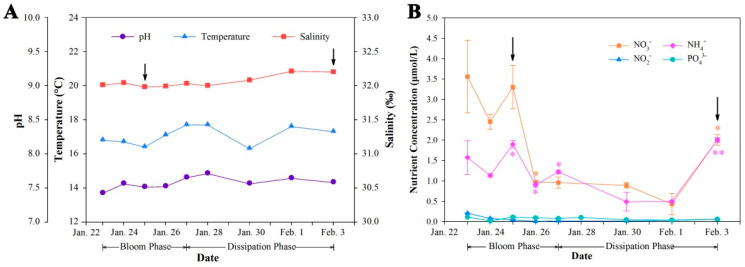
Physicochemical information for the *Phaeocystis globosa* bloom. (**A**) Physical oceanographic parameters. (**B**) Variation in inorganic nitrogen and phosphate concentrations. Values are reported as the means of triplicates with standard deviations. * (*p* ≤ 0.05) and ** (*p* ≤ 0.01) represent significant differences compared to the previous result. Arrows represent the sampling of proteomic study.

According to the physical oceanographic parameter measurements, the pH and salinity of seawater remained stable throughout the whole investigation (Figure 2A). Seawater temperature fluctuated between 16.3 and 17.7 °C, with an average of 17.1 °C, which is a favorable temperature for *P. globosa* bloom formation, according to previous studies [1,7].

Inorganic phosphate remained stable at a low level (0.074 ± 0.03 μmol/L) (Figure 2B), which was much lower than the optimal concentration (1 μmol/L) of phosphate for colony formation described in prior findings [23]. The inorganic nitrogen was dominated by nitrate and ammonium, and these two nitrogen forms presented similar dynamic profiles with a sharp decrease (*p* ≤ 0.05 for both nitrate and ammonium) on 26 January and a significant increase (*p* ≤ 0.05 for nitrate and *p* ≤ 0.01 for ammonium) on the last two days of the survey (Figure 2B).

### 2.3. Protein Identification and Differentially Abundant Proteins

The mass spectrometry experiment output 585,523 spectra, 45,941 of which were matched to 18,773 peptides. Using the MASCOT search engine, 5540 proteins were identified from 16,739 unique peptides derived from the matched spectra. Functional annotation of these identified proteins based on KEGG and NCBInr databases is presented in Table S1, and all these mass spectrometry proteomics data have been deposited to the ProteomeXchange Consortium (http://proteomecentral.proteomexchange.org, accessed on 5 September 2022) via the iProX partner repository [24] with the dataset identifier PXD036496.

Of the 5540 proteins identified, 300 proteins were differentially abundant, 191 were more abundant in the bloom phase, and 109 displayed higher abundances in the dissipation phase. The functional classification of these differentially abundant proteins is presented in Table S2.

**Figure 3:**
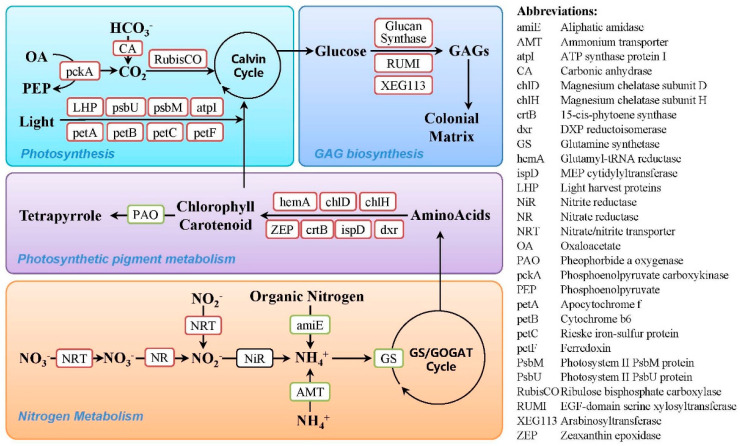
A schematic diagram depicting the functional distribution of the differentially abundant proteins between the bloom and dissipation phases. Proteins in red frame represent more abundance in the bloom phase and proteins in green frame represent more abundance in the dissipation phase.

According to the functional analysis, proteins with higher abundances in the bloom phase were dominated by proteins involved in photosynthesis. These include proteins involved in light harvest and electron transportation, such as light harvesting proteins, photosystem II proteins, ferredoxin and cytochrome, and three proteins participating in carbon fixation: ribulose bisphosphate carboxylase (RubisCO), carbonic anhydrase (CA), and phosphoenolpyruvate carboxykinase (pckA) (Figure 3). Consistently, the proteins involved in photosynthetic pigment biosynthesis were also more abundant in the bloom phase, while the protein pheophorbide *a* oxygenase (PAO), which regulates chlorophyll catabolism, displayed higher abundance in the dissipation phase (Figure 3). For nitrogen metabolism, nitrate/nitrite transporter (NRT) and nitrate reductase (NR) were more abundant in the bloom phase, while ammonium transporter (AMT), glutamine synthetase (GS), and another aliphatic amidase displayed higher abundances in the dissipation phase (Figure 3). Additionally, four proteins involved in glycosaminoglycan biosynthesis and a large number of transporters and membrane-trafficking proteins also displayed higher abundance in the bloom phase (Figure 3). In addition to these proteins with clear functions, it is noteworthy that approximately 40% of the differentially abundant proteins could not be annotated with any clear functions in either the KEGG or the NCBInr database (Table S2). These proteins likely contain important information related to algal bloom formation and should be further explored in future studies.

## 3. Discussion

In this study, a TMT-based quantitative proteomic approach was utilized to analyze the key intracellular biological processes underlying a *P. globosa* bloom. Hundreds of *P. globosa* proteins were differentially abundant between the bloom and dissipation phases. These proteins participate in a variety of biological processes, such as photosynthesis, nitrogen metabolism and GAG biosynthesis, and are closely associated with bloom development.

### 3.1. Photosynthesis

Functional analysis showed that proteins with higher abundances in the bloom phase were dominated by photosynthesis-related proteins, and these proteins covered both light-dependent and light-independent reactions of photosynthesis. Among them, light- harvesting proteins were components of the light-harvesting complex; additionally, photosystem II proteins, ferredoxin and cytochrome were proteins involved in light harvest and electron transportation. Higher abundances of these proteins suggest a greater energy production potential in blooming colonies. Moreover, it is noteworthy that ribulose bisphosphate carboxylase (RubisCO), carbonic anhydrase (CA), and phosphoenolpyruvate carboxykinase (pckA) displayed higher abundances in the bloom phase. These proteins mainly participate in light-independent reactions of photosynthesis and are the key enzymes of inorganic carbon-concentrating mechanisms (CCMs).

It is estimated that the rate of carbon fixation of *Phaeocystis* can reach 40 g carbon/(m^2^·month) during the bloom period, and the high biomass is actually one of the prominent features of *P. globosa* blooms [25]. However, the slow diffusion of CO_2_ actually limits inorganic carbon accumulation from seawater, and the affinity of RubisCO for CO_2_ cannot match the high rates of photosynthesis [26]. To overcome this limitation, marine phytoplankton enhance RubisCO production in low-CO_2_ environments, have evolved high-CO_2_-affinity RubisCO and, most importantly, have evolved CCMs, such as CA and C_4_ CCM [26,27]. Previous studies confirmed that CA is an important CCM enzyme in *P. globosa* [28]. It catalyzes the conversion of HCO_3_^−^ to CO_2_, which then diffuses across the membrane to support photosynthesis and growth. This CA CCM benefits *P. globosa* in interspecific competition, especially in situations of pH increase and CO_2_ decrease [29,30]. The protein pckA is one of three representative enzymes involved in the C_4_ CCM of phytoplankton [27]. Higher abundances of these proteins, therefore, indicate that *P. globosa* possesses multiple CCMs, which may explain why the inorganic carbon acquisition of *Phaeocystis* is equal to or more efficient than that in diatoms [28]. Overall, these results suggest that the inorganic carbon acquisition in blooming colonies is more efficient, and this should be a key factor used to support the massive biomass production of *P. globosa* blooms.

In addition, the variation in photosynthetic efficiency might be associated with the different vertical distributions of colonies between the bloom and dissipation phases (Figure S1). A previous laboratory experiment using *Phaeocystis* demonstrated that colonies could regulate their buoyancy according to the ambient light level, which conferred an advantage when accessing nutrients [17,18]. This light-dependent regulation is further suggested to be closely related to photosynthetic activity, since photosynthesis governs the amount of intracellular storage and the interior fluid chemistry of colonies, such as pH and oxygen concentration [31,32]. In this study, the differential abundances of photosynthetic proteins resulted in photosynthetic efficiency variation, which should partially account for the sinking of senescent colonies in the dissipation phase, which was also observed in other field investigations of *Phaeocystis* blooms [33].

### 3.2. Photosynthetic Pigment Metabolism

Consistent with the higher photosynthetic efficiency, proteins involved in photosynthetic pigment biosynthesis also displayed higher abundances in the bloom phase. As the major photosynthetic pigments, chlorophyll and carotenoids play essential roles in photosynthetic light harvesting and energy transduction in marine phytoplankton [34]. Chlorophyll is synthesized in three steps: the rate-limiting and committed step of 5-aminolevulinic acid formation, protoporphyrin IX biosynthesis, and chlorophyll production in the magnesium branch [35,36]. According to the functional classification, five differentially abundant proteins were involved in pigment biosynthesis, and all of them were more abundant in the bloom phase. These include glutamyl-tRNA reductase (hemA) participating in the first rate-limiting step of chlorophyll biosynthesis; magnesium chelatase subunit D (chlD) and subunit H (chlH) catalyzing protoporphyrin to protochlorophyllide, which belongs to the third step; and zeaxanthin epoxidase (ZEP) and phytoene synthase (crtB) participating in carotenoid biosynthesis [37]. In addition, another two proteins involved in biosynthesis of the substrate terpenoid backbone were more abundant in the bloom phase, which was coordinated with the higher abundances of pigment biosynthetic proteins [37]. Conversely, the protein pheophorbide *a* oxygenase (PAO), which regulates chlorophyll catabolism, displayed significantly higher abundance during the dissipation phase. PAO is one of the two proteins catalyzing the degradation of chlorophyll to colorless linear tetrapyrroles, and its expression is positively correlated with cell senescence [38]. The higher abundance of PAO, therefore, suggests that pigment degradation was enhanced in the senescent dissipation phase. Overall, these results indicated that enhanced pigment biosynthetic activity is essential for *P. globosa* blooms since it supports the high energy transduction and carbon fixation of blooming colonies.

### 3.3. Nitrogen Metabolism

Nitrogen is an essential nutrient for phytoplankton because of its contribution to building chlorophyll, amino acids, and nucleic acids. Laboratory experiments have demonstrated that nitrogen regulates the cell growth and colony formation of *Phaeocystis* colonies, and thus mediates field bloom processes [20,21,22]. In marine phytoplankton, ambient nitrate and nitrite are first transported into cells by nitrate/nitrite transporters (NRTs). The nitrate is then reduced to nitrite via nitrate reductase (NR), and nitrite is reduced to ammonia by nitrite reductase (NiR). The produced ammonia is finally incorporated into glutamine through the glutamine synthetase (GS)/glutamate synthase (GOGAT) pathway and then used for the biosynthesis of amino acids and other nitrogenous compounds [39].

In this study, NRT and NR were more abundant in the bloom phase, while an ammonium transporter (AMT) and GS displayed higher abundances during the dissipation phase, which indicated that nitrate utilization was more active during the bloom phase and that ammonium assimilation was enhanced during the dissipation phase of the bloom. This result was consistent with the nutrient investigation that showed a sharp decrease in the nitrate concentration during the bloom period (Figure 2B). Differential photosynthetic activity between these two phases should partially account for the varied abundances of these proteins since photosynthesis and nitrogen metabolism are integrally coupled and the nitrate metabolism of phytoplankton is mainly conducted in chloroplasts [40]. Moreover, previous studies revealed that nitrate is preferred for colony formation and *Phaeocystis* usually blooms in environments where nitrate is the major nitrogen source, while ammonium supports the growth of solitary cells rather than colonial cells [22,41]. Our proteomic results, therefore, support these findings and suggest that nitrate is a key ambient trigger of *P. globosa* blooms and that the enhanced nitrate utilization of colonial cells is essential for bloom maintenance.

In addition, it is noteworthy that a protein aliphatic amidase (amiE), which is involved in organic nitrogen metabolism, was differentially more abundant in the dissipation phase. This enzyme participates in short-chain aliphatic amide hydrolysis and produces the corresponding organic acids and ammonia, which provides the cell acetamide as both a carbon and a nitrogen source [42]. It helps phytoplankton recycle intracellular organic nitrogen and is usually upregulated under nitrogen-deficient conditions [43]. Moreover, in a study of *Emiliania huxleyi*, which is a phytoplankton species related to *P. globosa*, ambient amides were confirmed to be able to be transported into the cell and subsequently degraded to ammonia by aliphatic amidase [44,45]. The higher abundance of aliphatic amidase, therefore, indicates an enhancement of organic nitrogen utilization in colonies in the dissipation phase, either from intracellular nitrogen recycling or from extracellular organic substrates, such as the mucilaginous matrix of colonies. Overall, differential abundances of the nitrogen metabolic proteins revealed two distinct nitrogen utilization strategies between the two phases, in which the preferred nitrate of the bloom phase was converted to ammonium as the bloom developed to the senescent dissipation phase.

### 3.4. Glycosaminoglycan Biosynthesis and Transportation

For *Phaeocystis*, the colony morphotype is arguably the most salient feature because of its exceptional physiology and ecological advantages in interspecific competition [8]. In *Phaeocystis* colonies, thousands of cells are distributed evenly within the gelatinous matrix by which the colonies can dominate the phytoplankton community or even the entire ecosystem with massive biomass production during the bloom period [46,47].

Biochemical analysis confirmed that the gelatinous matrix of colonies is synthesized by the combination of a large number of cell-secreting GAGs [46]. For marine phytoplankton, the GAG polysaccharide biosynthesis process can be divided into three parts: the production of the sugar precursor, the chain polymerization of GAG, and the export of GAG to the cell surface [48]. In our study, four of the differentially abundant proteins participated in GAG biosynthesis, and all of them exhibited higher abundances in the bloom phase. These include arabinosyltransferase (XEG113), EGF-domain serine glucosyl/xylosyltransferase (RUMI), and two 1,3-beta-glucan synthases. All these proteins are glycosyltransferases and perform a similar GAG-chain-polymerization function that transfers the monosaccharide unit from the activated UDP-sugar precursors to assemble the GAGs [48,49]. The higher abundances of these proteins suggest that the biosynthesis of GAGs was more active in the bloom phase. This result agrees with the enhanced photosynthesis of the bloom phase since the majority of carbon fixed in *Phaeocystis* was released in the form of GAGs [25]. After being synthesized, GAGs are exported by *Phaeocystis* cells via exocytosis, in which a condensed polymer matrix first packs the output products, then encapsulates them in secretory granules, and finally releases them from the cell [25]. Further analysis indicated that this exocytosis process was stimulated by blue light and transduced by a characteristic intracellular Ca^2+^ signal [25]. Therefore, it is not surprising that a large number of transporters and membrane-trafficking proteins also exhibited higher abundances in the bloom phase (Table S2), which might participate in GAG exportation and support *P. globosa* colony formation.

## 4. Materials and Methods

### 4.1. Investigation and Sampling of Phaeocystis globosa Blooms

**Figure 4:**
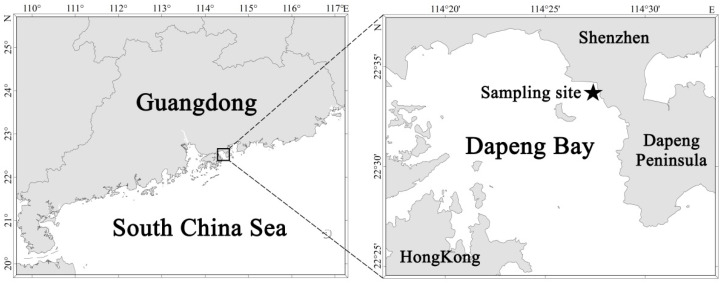
Sampling site (star) of *Phaeocystis globosa* bloom investigation in Dapeng Bay.

In 2021, a *P. globosa* bloom was traced in Dapeng Bay along the Shenzhen coast (22°33.6428′ N, 114°27.6264′ E) from 23 January to 3 February (Figure 4). 

During the field investigation, a cruise was conducted at a fixed time (10:00–11:00 a.m.) every day from 23 to 28 January and every two days for the last period. For each survey, 3 × 1 L surface water (0.5 m) was collected for colonial density monitoring. Large *P. globosa* colonies were identified and counted in situ, while small colonies were examined in culture dishes using a stereomicroscope (S9D, Leica, Germany) in the laboratory. Physical oceanographic parameters of seawater (pH, temperature, and salinity) were measured in situ with a YSI meter (Professional Plus 6600, YSI, Inc., Yellow Springs, OH, USA). Additionally, to investigate the colonial movement during the bloom period, a GoPro camera (GoPro HERO 7, GoPro, Inc., San Mateo, CA, USA) was utilized to capture the vertical distribution of the colonies from the surface to the bottom at the sampling site. 

For nutrient concentration, seawater was first filtered through a 0.45-μm membrane (Millipore). Then, the inorganic nitrogen and phosphate levels were determined using the diazo coupling method and phosphomolybdenum blue spectrophotometric method, respectively [50]. 

For proteomic analysis, colonies of surface water were collected in situ using a sampling mesh, transferred to petri dishes with a dropper pipette, rinsed with sterilized seawater, and frozen immediately with liquid nitrogen before transfer to −80 °C storage in the laboratory.

### 4.2. Protein Preparation and Mass Spectrometry

According to the field investigation results and a previous study of the same *P. globosa* bloom [51], the colony density of *P. globosa* reached the highest level on 25 January and started to dissipate from 27 January. Therefore, in this study, samples from 25 January were selected as the bloom phase (BP), and those from 3 February were selected as the dissipation phase (DP) of the *P. globosa* bloom. Three biological replicates were used to compare the proteomes of these two phases.

Proteins were extracted using TRIzol reagent following a previously reported protocol and quantified with the Bradford assay, in which bovine serum albumin (BSA) was used as a standard [52]. After extraction, a filter-aided sample preparation procedure was used for trypsin digestion of the proteins [53]. A total of 100 μg of peptide mixture from each sample was labeled using TMT reagent according to the manufacturer’s instructions (Thermo Fisher Scientific, Waltham, MA, USA). The TMT-labeled digest samples were then fractionated into 10 fractions using a high-pH reversed-phase fractionation kit (Thermo Scientific, Waltham, MA, USA), and the collected fractions were desalted with C18 cartridges (Empore™ SPE Cartridges C18, Sigma, Burlington, MA, USA). Finally, all the desalted fractions were concentrated for subsequent mass spectrometry using vacuum centrifugation.

LC-MS/MS analysis was performed for 90 min with a Q-Exactive mass spectrometer (Thermo Scientific, Waltham, MA, USA) coupled to an Easy nLC instrument (Proxeon Biosystems, Foster City, CA, USA). Positive ion mode and peptide recognition mode were set for the mass spectrometer operation. MS data acquisition was conducted using the data-dependent top 10 method, in which the most abundant precursor ions of the survey scan at 300–1800 *m*/*z* were dynamically chosen for subsequent higher-energy collisional dissociation (HCD) fragmentation. During this process, the parameters were set as follows: automatic gain control (AGC) target at 3 × 10^6^, maximum injection time of 10 ms, dynamic exclusion duration for 40.0 s, survey scan resolution of 70,000 at 200 *m*/*z*, HCD spectrum resolution of 17,500 at 200 *m*/*z*, isolation width of 2 *m*/*z*, and normalized collision energy of 30 eV. In addition, the underfill ratio that specifies the minimum percentage of the target value likely to be reached at maximum fill time, was defined as 0.1%.

### 4.3. Bioinformatics Analysis

The output MS/MS spectra were searched using the MASCOT search engine (Matrix Science, London, UK; version 2.2) embedded in Proteome Discoverer 1.4. Protein identification was performed using a self-built database consisting of 61,148 peptide sequences that translated the sequences of unigenes from a previous transcriptomic study of *P. globosa* [16]. All peptide sequences were sorted to a FASTA format file (24.2 MB) before the searching process.

For protein identification, the search parameters were set as follows: fragmented ion mass tolerance of 0.1 Da, peptide mass tolerance of 2 × 10^−5^ with allowance for up to two missed cleavages. Fixed modifications were set as TMT labeling and carbamidomethylation, and a potential variable modification was set as oxidation (M). In addition, an automatic decoy database search was performed in MASCOT to estimate the false discovery rate, in which random sequences were generated and then tested for spectra in the decoy database and the real database. A protein was considered identified if it contained at least one unique peptide.

For protein functional annotation, the amino acid sequence of each protein was searched against the Kyoto Encyclopedia of Genes and Genomes (KEGG) and National Center Biotechnology Information nonredundant protein sequences (NCBInr) databases using BLAST client software.

For protein quantitation, the protein ratios were calculated as the median values of their corresponding unique peptides. Protein abundances were compared in DP/BP, and the average ratio of three replicates was calculated as the differential protein abundance. A protein was considered to be differentially abundant if it had a significance ratio (t test *p* value ≤ 0.05) of DP/BP ≥ 1.5 (higher abundance in the DP group) or DP/BP ≤ 0.67 (i.e., BP/DP ≥ 1.5, higher abundance in the BP group).

## 5. Conclusions

Our study investigated a *P. globosa* bloom that occurred along the Chinese coast and, for the first time, studied the key intrinsic biological processes associated with the in situ bloom process using a TMT-based quantitative proteomic approach. Proteins involved in photosynthetic processes, pigment biosynthesis, nitrate utilization, and matrix substrate biosynthesis displayed higher abundances during the bloom phase. The colonies in the bloom phase have greater carbon fixation potential, and more carbon and energy are fixed and flow to the matrix biosynthesis. Ambient nitrate is a key trigger of *P. globosa* bloom formation, while enhanced light harvest and multiple CCMs support prosperous colonies in the bloom phase. Our study revealed the key biological processes underlying the blooming of *P. globosa* and shed new light on the mechanisms driving the formation of *P. globosa* blooms.

## Data Availability

The mass spectrometry proteomics data have been deposited in the ProteomeXchange Consortium (http://proteomecentral.proteomexchange.org, accessed on 5 September 2022) with the dataset identifier PXD036496.

## Supplementary Materials

The following supporting information can be downloaded at: https://www.mdpi.com/article/10.3390/ijms232012668/s1.
