# Supplementary figures and images for: Quantitative Proteomic Analysis Reveals the Key Molecular Events Driving Phaeocystis globosa Bloom and Dissipation
Source: Int J Mol Sci. 2022 Oct 21;23(20):12668. doi: 10.3390/ijms232012668 (PMC9604223; doi:10.3390/ijms232012668)

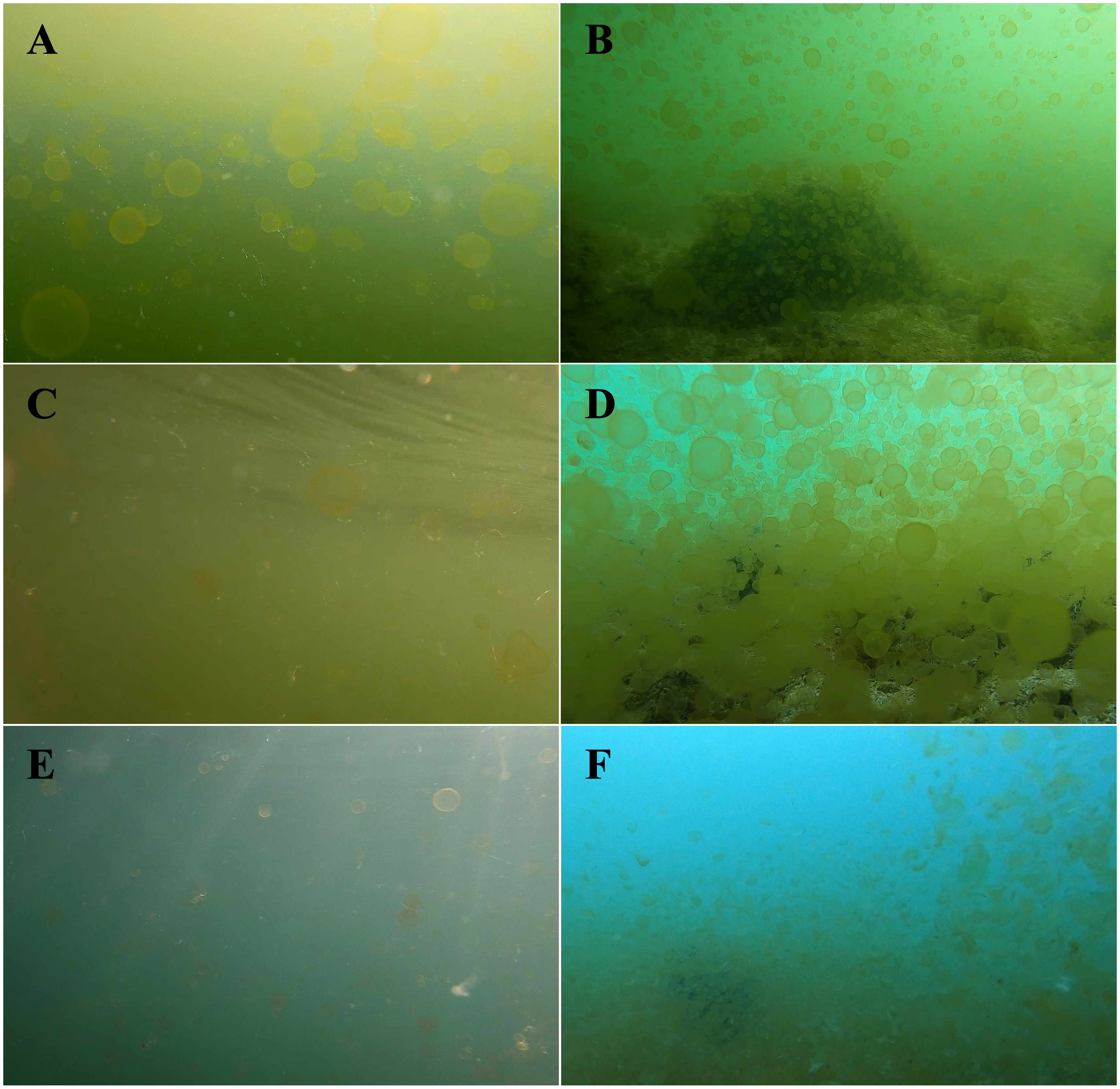

Supplement: Supplementary file 1 [file ijms-23-12668-s001.zip › Supplemental Figure S1.jpg]
